# A Technique for Dissecting the Salivary Glands From the Abdomens of Deer Keds (Diptera: Hippoboscidae: *Lipoptena* Nitzsch, 1818 and *Neolipoptena* Bequaert, 1942)

**DOI:** 10.1093/jisesa/ieaa027

**Published:** 2020-11-02

**Authors:** Michael J Skvarla, Karen C Poh, Jesse R Evans, Erika Machtinger

**Affiliations:** Department of Entomology, Penn State University, University Park, PA

**Keywords:** deer ked, hippoboscid, dissection, salivary gland, pathogen screening

## Abstract

Deer keds (Diptera: Hippoboscidae: *Lipoptena* Nitzsch, 1818 and *Neolipoptena* Bequaert, 1942) are hematophagous ectoparasites of cervids that occasionally bite other mammals, including humans. In recent years, a number of arthropod-borne pathogens have been sequenced from deer keds. However, it is unclear if the pathogens are just present in host blood in the gut or if the pathogens are present in other organs (e.g., salivary glands) that would suggest that keds are competent vectors. Like other hippoboscoid flies, deer keds have extensive salivary glands that extend through the thorax and into the abdomen, so simply disarticulating and sequencing the thorax and abdomen separately does not circumvent the issues surrounding whole-body sequencing. Herein, we describe a technique for dissecting the terminal portion of the salivary glands from the abdomen in order to screen the thorax and salivary glands separately from the abdomen for arthropod-borne pathogens.

Deer keds (Diptera: Hippoboscidae: Lipopteninae: *Lipoptena* Nitzsch, 1818 and *Neolipoptena* Bequaert, 1942) (Fig. 1) are hematophagous ectoparasites of cervids (Artiodactyla: Cervidae) that occasionally bite other mammals, including humans (Skvarla and Machtinger 2019). Thirty-two species are described worldwide, four of which occur in North America: *Lipoptena cervi* (Linnaeus, 1758) (northeastern United States and Canada), *L. mazamae* Róndani, 1878 (southeastern United States south through Brazil), and *L. depressa* (Say, 1823) and *Neolipoptena ferrisi* Bequaert, 1942 (western United States and Canada) (see Figure 3 in Skvarla and Machtinger 2019 for a range map).

**Fig. 1.**
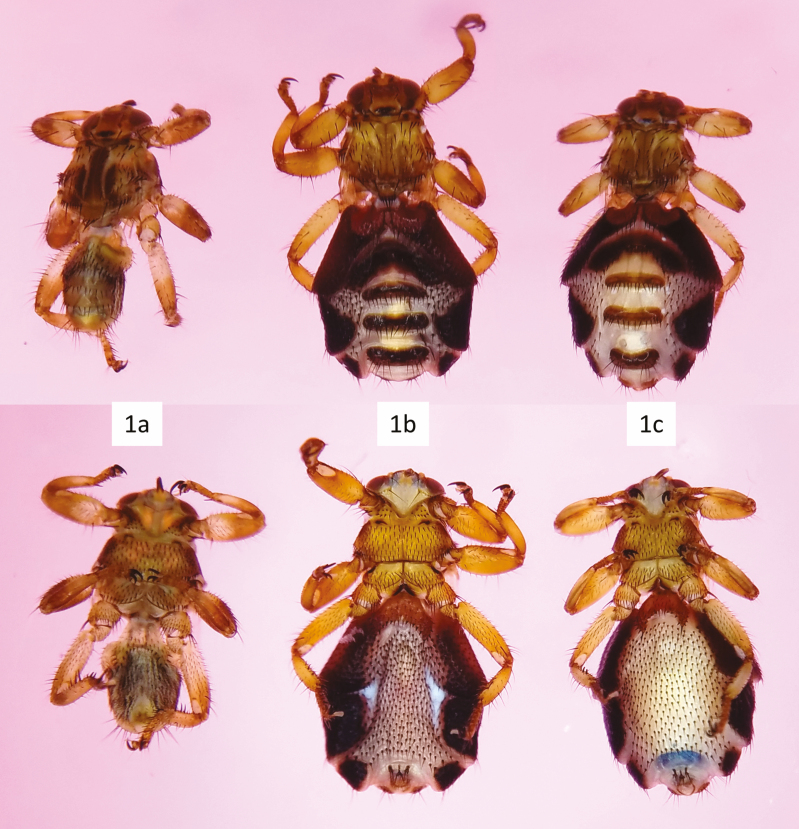
Deer keds (*L. cervi*). Top row: dorsal. Bottom row: ventral. (a) Unfed ked with small abdomen. (b) Ked that has fed at least once with an expanded abdomen. (c) Ked with mature larva visible through the ventral abdomen.

Deer keds have historically been regarded as pests of only minor veterinary and medical importance. However, in Europe, reindeer (*Rangifer t. tarandus* (Linnaeus, 1758); Artiodactyla: Cervidae) exhibit increased head shaking, grooming, and other stress-related signs during ked infestations (Kynkäänniemi et al. 2014) and moose (*Alces alces* Linnaeus, 1758; Artiodactyla: Cervidae) in northern Europe have developed hair loss and decline in condition during extremely heavy infestations (Madslien et al. 2011). Similar conditions have been less studied in North America, although Kellogg et al. (1971) observed that keds are a ‘constant annoyance to wild deer’ and Heine et al. (2017) showed that keds may be more influential on deer-grooming behavior than ticks. In northern Europe, deer keds can become so numerous that they are associated with ked bite dermatitis and considered an occupational hazard (Chistyakov 1968, Laukkanen et al. 2005, Härkönen et al. 2009). Similar ‘hotspots’ of deer ked abundance and activity have been anecdotally reported by hunters in North America but do not appear to be as widespread a phenomenon.

Deer keds have not been considered vectors of arthropod-borne pathogens historically (Skvarla et al. 2019). However, in recent years, a number of tick-borne pathogens have been sequenced from deer keds, including *Anaplasma* (Rickettsiales: Anaplasmataceae) (e.g., de Bruin et al. 2015, Buss et al. 2016), *Bartonella* (Rhizobiales: Bartonellaceae) (e.g., de Bruin et al. 2015, Izenour et al. 2020), and *Rickettsia* (Rickettsiales: Rickettsiaceae) (e.g., Hornok et al. 2011, de Bruin et al., 2015) (see Table 1 in Skvarla and Machtinger (2019) for a list of pathogen publications). It has even been suggested that the aforementioned ked-associated dermatitis may be caused by a ked-vectored *Bartonella* species (Dehio et al. 2004).

To date, every study that has screened adult deer keds for pathogens has been based on whole-body extractions (see Table 1 in Skvarla and Machtinger (2019) for a list of publications). It is thus unclear if the pathogens detected in positive samples are from host blood contained in ked digestive tracts or if they are present in other organs, such as the salivary glands (e.g., Baker 1967, Klei and Giusti 1973, Ueti et al. 2007, Lejal et al. 2019), which might support the idea that keds are competent pathogen vectors. One possible solution would be to screen the digestive tract, and any host blood it might contain, separately from the rest of the body, and it might seem intuitive to simply separate the head and thorax from the abdomen, which contains the majority of the digestive tract. However, deer keds have extensive salivary glands that extend through the thorax into the anterodorsal area of the abdomen (Figure 15 in Bequaert 1953), which would confound such efforts. While there are portions of the digestive tract in the thorax, they are not used to store blood meals and unlikely to contain host blood unless a deer ked was collected during or immediately after feeding—the crop is the largest section of the digestive tract in the thorax, but Bequaert (1953) found that it does not fill with or store blood in *Lipoptena,* unlike in the bird-feeding keds (Ornithomyinae), and the portion of the diverticulum that is used to store extra blood is confined to the abdomen (Theodor 1928, Tarshis 1957).

Because deer keds are small, highly sclerotized, dorsoventrally flattened ectoparasites and their salivary glands are soft, white organs attached to the mouthparts by a thin duct, it was not immediately obvious how to best separate the salivary glands from the abdomen. We found that attempting to access them through the dorsal abdominal cuticle was often not successful and devised the method for separation presented here, for which we provide step-by-step instructions and photomicrographs.

## Experimental Design

The tools we used to dissect deer keds are listed in Table 1. Manufacturer/vendor information is provided for each item; however, the exact materials listed do not need to be used. The first author learned how to create the dissection tools described here from Dr. Don Steinkraus at the University of Arkansas, who incorporated techniques previously suggested by Smith (1923), Dade (1962), and others.

**Table 1.** Materials used to dissect deer keds

Item	Manufacturer/vendor	Catalog/item number
Fine-point forceps	Bioquip	4535
Insect pins, #2	Bioquip	1208B2
Insect pins, #1	Bioquip	1208B1
Wax-bottom Petri dish	Custom made	N/A
Petri dish, glass, 100 × 20 mm	VWR	75845-514
Dental wax		N/A
Microscalpel	Custom made	N/A
Dissection probe	Custom made	N/A
Utility knife replacement blade	Workpro (Amazon)	N/A
Breakable scalpel blade	Fine Science Tools	10050-00
Blade breaker	Fine Science Tools	10052-11
Bamboo skewer	Amazon	N/A
Lineman’s pliers	Home Depot/Husky	48057
Diagonal cutting pliers	Home Depot/Husky	48056
Epoxy resin (ClearWeld)	Home Depot/JB Weld	50114H
70% ethanol (diluted from 100%)	Koptec	V1001
SZ-61 stereomicroscope (0.75–13.5 magnification)	Olympus	SZ-6145
Iris scossors	Bioquip	4715

**Fig. 2.**
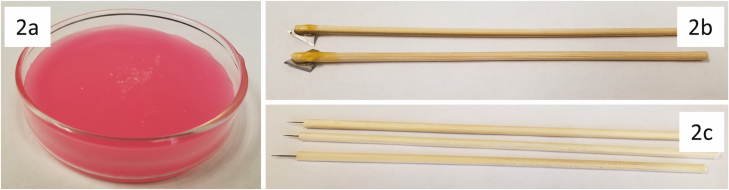
Dissection tools. (a) Petri dish with wax bottom. (b) Microscalpels. (c) Probes.

Wax-bottomed Petri dishes (Fig. 2a) were made by melting dental wax in a double-boiler (an empty food can in a pot of boiling water) on a kitchen stove and pouring the hot wax into a 90 × 20 mm glass Petri dish, which was then allowed to cool overnight. Unlike pure paraffin wax, dental wax was used because it is stiff but not brittle. If dental wax is unavailable, a 1:1 mix of paraffin and beeswax can also be used.

Microscalpels and dissection probes (Fig. 2b) were manufactured using bamboo skewers, which were cut to a comfortable length (15–20 cm), microblades, and insect pins. Microscalpel blades were manufactured by securing a utility knife blade in a vice and breaking a corner from the blade using lineman’s pliers. A small split was made in a bamboo skewer using a utility knife and the scalpel blade was pushed into the split using lineman’s pliers and affixed with epoxy. This technique resulted in somewhat ununiform microscapels but was easily done with materials available at hand. If more uniform blades are desired, breakable scalpel blades and blade breakers are available.

Dissection probes were made by cutting off the apical 1–2 cm of an insect pin using diagonal cutting (or side-cutting) pliers and pushing the cut pin into a bamboo skewer using lineman’s pliers. Cutting different sized pins (#0–3) and creating multiple probes of various sizes is recommended. Probes manufactured using #0 pins were found to dull after several dissections, so if many dissections are planned, it may be worthwhile to use sharpenable tungsten wire probes (for materials and methods, see Brady 1965).

## Protocol

The protocol described here is best performed on wingless deer keds that have fed at least once (Fig. 1a) and have been stored in 70–80% ethanol. Such keds are most easily collected from host animals. Keds that have not fed, such as winged specimens that land on people, have much smaller abdomens (Fig. 1b). Because there is no risk of sequencing pathogens from host blood in unfed keds, the dissection outlined here is unnecessary for pathogen screening; however, unfed keds are more difficult to collect in large quantities. Specimens stored in higher percentage ethanol or isopropanol can become brittle and break easily during dissection.

**Fig. 3.**
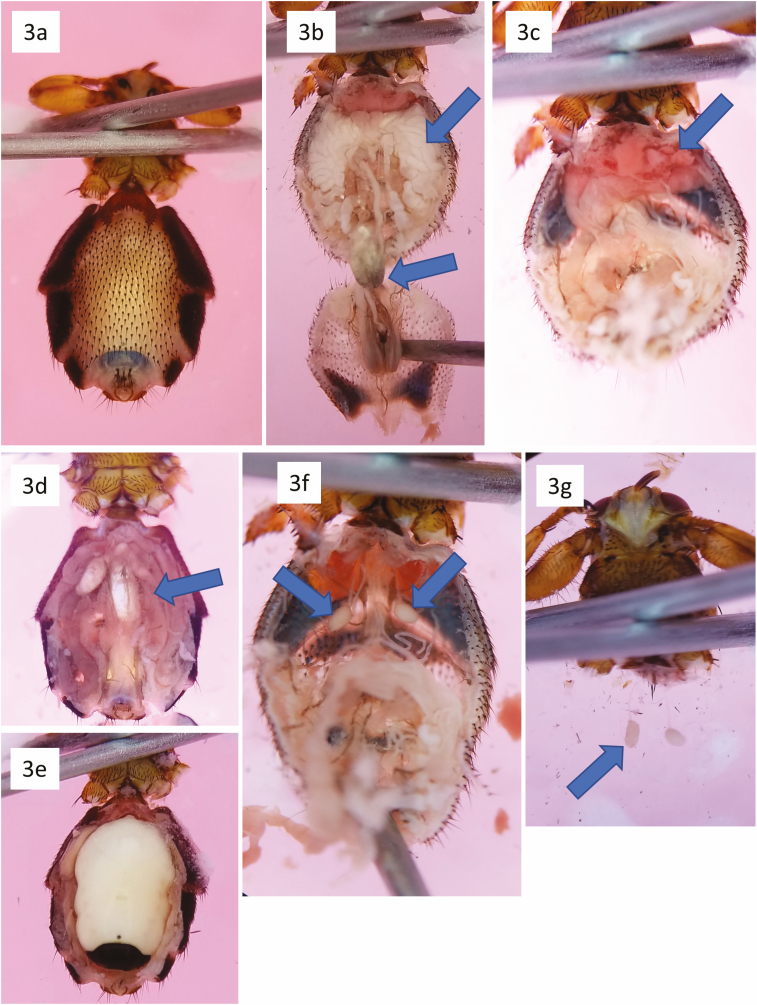
Abdominal and salivary gland dissection process. (a) Ked with mid- and hind legs removed. (b) Ventral abdomen removed and pulled back. Specimen is a male, note the aedeagus and extensive accessory glands. (c) Male specimen with accessory glands removed. Note the blood-filled portion of the anterior digestive tract. (d,e) Female keds with a developing larva (indicated by arrow) and a fully mature larva. (f) Abdomen after the digestive tract has been removed. Salivary glands are indicated by arrows. (g) Ked post-dissection after salivary glands (indicated by arrows) have been isolated and abdomen has been removed.

Begin by adding enough 70% ethanol to the wax Petri dish to fully submerge a deer ked, but not so much that it splashes if you move the dish. Transfer a ked to the Petri dish and remove the middle and hind legs using iris scissors or a microscalpel. Once the legs are removed, secure the ked to the wax ventral-side up using two #2 or #3 pins across the thorax just behind the front legs (Fig. 3a). Once the ked is secured in position, hold the abdomen in place with forceps and cut the cuticle along the lateral edge; the cuticle of the first ventrite is more sclerotized, but can be carefully cut with a microscalpel. The juncture of the thorax and abdomen is thin, so be sure not to cut completely through the abdomen when working that area. The entire venter can be cut away and removed or a small flap left along the posterior so that it can be pulled back like a flap (Fig. 3b).

Next, use two probes to gently loosen and remove the posterior half of the digestive tract. If the digestive tract is empty, it should remain malleable and come out intact (Fig. 3b). If the ked fed before being collected, there may still be partially digested blood in the digestive tract. If a bloodmeal is present (Fig. 3c), the digestive tract may be difficult to remove intact; however, if it breaks apart and blood is released, the blood should appear as solid clumps that can be pushed aside with a probe.

Because deer keds reproduce via adenotrophic viviparity, eggs and partially to fully developed larvae may be encountered (Fig. 3d and e). Fully developed larvae can often be seen through the cuticle prior to dissection (Fig. 1c). Eggs and larvae can be dissected from the uterus for separate pathogen screening if desired. Mature larvae are especially easy to remove as they are large and the membrane surrounding them is thin. However, because mature larvae take up so much space in the abdomen and consequently compact the surrounding viscera, it is often difficult to find and isolate the salivary glands without destroying the glands.

Once any eggs or larvae, testes and accessory glands, and most of the digestive tract have been removed, slowly tease apart the remaining parts of the anterior digestive tract. The two salivary glands should appear as white sac-shaped organs attached to thin ducts that enter the thorax (Fig. 3f). The glands are often appressed to the dorsal parts of the abdomen, so they may need to be gently separated from the abdomen. Once the salivary glands and ducts are free, slide a microscalpel between the glands and abdomen and cut the abdomen free of the thorax (Fig. 3g). During the dissection, keep in mind that it is often easier to rotate the Petri dish to achieve the appropriate angle with a microscalpel or probe rather than contorting the hand and wrist.

With steady hands, patience, and a bit of luck, the salivary glands should now be free of the abdomen and majority of the digestive tract but still attached to the thorax. The dissected parts of the abdomen and head, thorax, and salivary glands can be transferred to separate containers for pathogen screening or other work. Occasionally one or both glands may be broken off so only the ducts remain. When this happens, it is sometimes possible to find the broken salivary gland amongst the digestive tract and extract it so that it can be processed separately from the digestive tract.

## Discussion

With practice, the lead author was able to perform the described dissection in 8–10 min per specimen and consistently recover the salivary glands intact. Other methods that were attempted (e.g., cutting through the dorsal abdomen) may be slightly faster, but did not reliably produce intact salivary glands.

Tarshis (1955) described and illustrated a similar method for dissecting bird keds (*Stilbometopa impressa* (Bigot, 1885) and *Icosta hirsuta* (Ferris, 1927)), which we independently arrived upon while dissecting out the salivary glands of deer keds. Much like how replication of experimental studies is needed to confirm the results of those studies, this ‘convergent evolution’ of techniques suggests that the techniques described by Tarshis (1955) and here are the most optimized way to dissect keds.

Bird keds are generally larger than deer keds, and the methods described by Tarshis (1955) are for finer, more precise dissections that likely take longer to perform. Because we are only interested in separating the salivary glands from the rest of the abdomen, the method presented here can be performed faster. Pairing the directions in the protocol section with the step-by-step photos will reduce the learning curve of those who attempt the described dissection for the first time.
